# A Century of Gibberellin Research

**DOI:** 10.1007/s00344-015-9546-1

**Published:** 2015-10-13

**Authors:** Peter Hedden, Valerie Sponsel

**Affiliations:** Rothamsted Research, West Common, Harpenden, AL5 2JQ Hertfordshire UK; Department of Biology, The University of Texas at San Antonio, San Antonio, TX 78249 USA

**Keywords:** Gibberellin biosynthesis, Gibberellin action, Gibberellin transport, Evolution, *Gibberella fujikuroi*

## Abstract

Gibberellin research has its origins in Japan in the 19th century, when a disease of rice was shown to be due to a fungal infection. The symptoms of the disease including overgrowth of the seedling and sterility were later shown to be due to secretions of the fungus *Gibberella fujikuroi* (now reclassified as *Fusarium fujikuroi*), from which the name gibberellin was derived for the active component. The profound effect of gibberellins on plant growth and development, particularly growth recovery in dwarf mutants and induction of bolting and flowering in some rosette species, prompted speculation that these fungal metabolites were endogenous plant growth regulators and this was confirmed by chemical characterisation in the late 1950s. Gibberellins are now known to be present in vascular plants, and some fungal and bacterial species. The biosynthesis of gibberellins in plants and the fungus has been largely resolved in terms of the pathways, enzymes, genes and their regulation. The proposal that gibberellins act in plants by removing growth limitation was confirmed by the demonstration that they induce the degradation of the growth-inhibiting DELLA proteins. The mechanism by which this is achieved was clarified by the identification of the gibberellin receptor from rice in 2005. Current research on gibberellin action is focussed particularly on the function of DELLA proteins as regulators of gene expression. This review traces the history of gibberellin research with emphasis on the early discoveries that enabled the more recent advances in this field.

## Introduction

The origins of gibberellin research can be traced to the late 19th century in Japan with the demonstration that a disease of rice that produced symptoms of excessive seedling elongation and infertility, among others, was the result of fungal infection (Hori 1898). Culture filtrates of the fungal pathogen were later shown to reproduce the symptoms in rice, and the active growth-promoting principle was named gibberellin after the perfect (reproductive) form of the fungus, *Gibberella fujikuroi*. Various names for the disease were used by Japanese farmers depending on location, the most well-known being “bakanae”, translated as silly seedling. The early research leading to the discovery, isolation and structural determination of gibberellins and the realisation that these compounds may be endogenous growth regulators in plants has been reviewed in detail by Phinney (1983). His review contains photographs of the principal scientists involved in this research. Phinney points out that while work on gibberellins before 1945 was restricted to Japan, some coverage of this research was available to the West in the 1930s through Chemical Abstracts, but did not inspire interest. However, following the 2nd World War, with freer communication with Japan, scientists in the USA and UK realised the importance of these compounds and active research programs were initiated in the 1950s. These and continuing work in Japan resulted in the isolation and structural determination of the main active compound from the fungus, named gibberellic acid in the UK and gibberellin-X in the USA, with the name gibberellic acid being agreed between them. The same compound was known as gibberellin A_3_ (GA_3_) in Japan.

Gibberellic acid was found to have profound effects on plant growth, with the ability to rescue dwarf mutants of maize and pea, and induce bolting and flowering in rosette species. These effects could also be obtained with plant extracts, providing a strong indication that gibberellins were endogenous plant metabolites. This was confirmed by the isolation of gibberellin A_1_ (GA_1_) from immature seeds of runner bean, *Phaseolus coccineus*, in 1958 (MacMillan and Suter 1958). Since this time there was steady progress in understanding gibberellin biosynthesis, the pathways being delineated in *G. fujikuroi* and higher plants by the 1970s, with the nature of the participating enzymes characterised for plants by the 1980s. With the cloning of the transcripts encoding these enzymes in the 1990s, the way was open to investigate how gibberellin metabolism is regulated, a topic of research that is still very active. Progress in understanding gibberellin action was initially slow, with much of the early work focused on the cereal aleurone, which responds to gibberellin by synthesising and secreting hydrolytic enzymes such as α-amylase. However, major breakthroughs in the 1990s and 2000s transformed our understanding of gibberellin function at the molecular level. With the cloning of the *GAI* cDNA in *Arabidopsis thaliana* (*Arabidopsis*) and its mutant allele *gai*, which produces GA-insensitivity, Peng and others (1997) suggested that gibberellins act to relieve growth repression by GAI (a member of the DELLA subgroup of the GRAS family of transcriptional regulators). The demonstration that gibberellin induces DELLA protein degradation via the ubiquitination–proteasome pathway, and the isolation of the GID1 GA receptor have enabled a detailed understanding of the early events in gibberellin perception and action. DELLA proteins are now known to act in partnership with transcription factors to regulate gene expression, and their function is currently an active area of research.

This article traces the major events in the gibberellin research timeline focussing particularly on the earlier work, which tends to become lost in the mists of time.

## Gibberellins as Fungal Metabolites: Early Research

The plant pathologist Kenkichi Sawada, working at the Imperial Research Institute at the Department of Agriculture in Taipei, Taiwan, was the first to suggest that the bakanae fungus provided the stimulus that caused the overgrowth symptoms in rice (Sawada 1912). This was later confirmed by his colleague Eiichi Kurosawa, who published a paper in 1926 showing that the symptoms of the disease could be reproduced by application of sterile fungal cultures (Kurosawa 1926). He found that the secreted “toxin” stimulated the growth of seedlings of several species besides rice. This landmark publication was followed by numerous reports on the properties of the secreted substances, and in 1935, the chemist Teijiro Yabuta, who was Professor of Agricultural Chemistry at the University of Tokyo, obtained a purified sample with high biological activity, which was called gibberellin after its fungal source (Yabuta 1935). Subsequently, the sample yielded two crystalline substances, which were named gibberellin A and gibberellin B (Yabuta and Sumiki 1938), with the names apparently being reversed in later publications. Although there were a number of reports on the chemical properties of these substances, they were later shown to be impure so these studies were inconclusive. It was not until the 1950s that chemists at Tokyo University, including Nobutaka Takahashi and Saburo Tamura, returned to the chemical nature of gibberellin A, and showed that it was a mixture of at least three compounds, which were isolated as their methyl esters and named gibberellin A_1_, gibberellin A_2_ and gibberellin A_3_ (Takahashi and others 1955). This system of nomenclature was later adopted for all gibberellins that were subsequently isolated (see below). The nature of gibberellin B is still unclear.

Research on gibberellins outside of Japan began in the 1950s when the Japanese work and its significance were finally appreciated in the West. It started at about the same time in the UK and USA. The British group led by Percy Brian at the ICI Akers Laboratories in Welwyn, north of London, was alerted to the early Japanese work by reports in Chemical Abstracts and began screening the ICI *Fusarium* collection for gibberellin production. Crystalline active preparations were passed for structural studies to the chemistry group, which was led by John Grove and included Jake MacMillan, Brian Cross, Philip Curtis and Paddy Mulholland. They were able to obtain a pure crystalline compound, which they called gibberellic acid (Curtis and Cross 1954). A structure for gibberellic acid was proposed in 1956, the evidence appearing in a series of papers, and reviewed by Grove (1961). An X-ray crystal structure for gibberellic acid as its di-*p*-bromobenzoate methyl ester was published in 1963 by Hartsuck and Lipscomb (1963).

Studies on gibberellin production in the USA were initiated by John Mitchell, a mycologist working at Camp Dietrick, Maryland. He procured a gibberellin-producing strain from Japan from which he was able to obtain growth-promoting extracts (Mitchell and Angel 1951). On Mitchell’s recommendation, a unit headed by Kenneth Raper was set up at the USDA laboratories in Peoria, Illinois, to produce gibberellin for agricultural trials. Further strains of the fungus were provided by Yusuki Sumiki, who had taken over as Professor of Agriculture at Tokyo University after the retirement of Yabuta in 1950. Sumiki presented the results of the Japanese research on gibberellins to the West, visiting the Peoria laboratories in 1951 and the Akers Laboratories in 1953. After initial difficulties, the Peoria group was able to produce good yields of gibberellin, and by 1953 under the leadership of the chemist Frank Stodola had obtained a pure crystalline product, gibberellin-X (Stodola and others 1955). As described above, gibberellin-X and gibberellic acid were found to be identical, and the name gibberellic acid was agreed on. This compound also proved to be identical with the Japanese gibberellin A_3_. Its availability opened the way for detailed studies on the effects of this fungal metabolite on plant growth and development.

## Gibberellins in Higher Plants

In the mid to late 1950s, numerous reports on the effects of gibberellin on plants appeared in the literature. Of particular note was the ability of gibberellic acid to rescue the growth defect in dwarf mutants of pea (Brian and others 1954; Brian and Hemming 1955) and maize (Phinney 1956) and to induce bolting and flowering in a number of biennial rosette species (Lang 1956; Wittwer and others 1957). At this time, auxin was the only known endogenous plant growth regulator, but the remarkable properties of gibberellins prompted the suggestion that they may also be naturally occurring in plants. The idea that dwarf peas may lack gibberellin prompted Margaret Radley, working with Percy Brian at the Akers laboratory, to apply extracts of tall peas to dwarf peas and demonstrate that they produced a similar growth response as gibberellic acid (Radley 1956). In similar experiments, Bernard Phinney and colleagues at UCLA used dwarf maize in bioassays to show that extracts from a number of plant species contained gibberellin-like substances (Phinney and others 1957). The first definitive evidence for the occurrence of gibberellins in plants was provided by Jake MacMillan and P.J. Suter, who isolated 2 mg of gibberellin A_1_ from 87.3 kg of immature seeds of runner bean (*Phaseolus multiflorus*, later reclassified as *Phaseolus coccineus*) (MacMillan and Suter 1958). They later identified gibberellins A_5_ (MacMillan and others 1959), A_6_ and A_8_ (MacMillan and others 1962) from the same source.

Following the first characterisation of gibberellins from runner bean, new gibberellins were isolated from different plant sources and given names in a rather haphazard fashion based on their plant origin, as, for example, bamboo gibberellin (Murofushi and others 1966) or Lupinus gibberellin-I (Koshimizu and others 1966). However, in the naming of new gibberellins such as A_4_ and A_7_ isolated from *Gibberella fujikuroi*, the numerical system that had been employed in Japan was continued. Furthermore, MacMillan and colleagues adopted this system in naming the gibberellins from *P. coccineus* seed. To put gibberellin nomenclature on a more systematic basis, it was agreed at the International Conference on Plant Growth Substances held in Ottawa, Canada, in 1967 that this numbering system would be used for all gibberellins, with Jake MacMillan and Nobutaka Takahashi assigning numbers to new gibberellins as they are identified (MacMillan and Takahashi 1968). After their retirement, this task was taken over by Yuji Kamiya and Peter Hedden. It is now common practice to abbreviate gibberellin A_x_ as GA_x_, with the generic abbreviation GA commonly used for gibberellin. It has led to the misconception that GA is an abbreviation of gibberellic acid, but as will be clear from the above discussion, gibberellic acid is a specific compound and is synonymous with GA_3_. As it turns out, GA_3_ is a minor gibberellin in higher plants.

Initially, structural characterisation of novel GAs required the isolation of large quantities of pure material, with structures based on chemical degradation to simpler compounds of known structure. As more chemically characterised GAs and related compounds became available, it was often possible to use conversion to known compounds in relatively few steps to confirm novel structures. Furthermore, the use of nuclear magnetic resonance reduced the required amounts of material to mg, and more recently μg quantities. However, these methods required the isolation of pure material, which, with concentrations of GAs in plant tissues often at levels of ng.g^−1^ fresh weight, is rarely feasible. The development of combined gas chromatography–mass spectrometry (GC–MS) for the analysis of GAs (and other plant metabolites) in MacMillan’s laboratory in the late 1960s offered new opportunities (Binks and others 1969). GC–MS was much more sensitive than other analytical methods available at the time and could be used with impure extracts. It was ideal for identifying known compounds for which mass spectra were available, although it could not be used to determine the structures of novel compounds directly. However, in many cases, characteristic fragmentation patterns allowed structures to be predicted, and the assumed structures could then be synthesised for GC–MS comparison with the native compound. By this means, the numerous novel GA-related structures synthesised in several chemistry labs, including those of Lewis Mander at the Australian National University, Canberra, Australia and Jake MacMillan at the University of Bristol, UK, have enabled the number of naturally occurring GAs to expand to 136. The source of the first 126 GAs in plants, fungi and bacteria was catalogued by MacMillan (2002). Many of these occur in developing seed at often high concentration, but their function is unknown. It is noteworthy that no new GA has been characterised in over 10 years, although further natural GAs must exist. This may be due in part to their structures not being easily synthesised, but also reflects the current lack of chemistry laboratories with an active GA program, with only Lewis Mander active in the field in recent years. His provision of isotopically labelled GAs for analytical and metabolism studies has been vitally important for GA research.

## Gibberellin Metabolism

### The Biosynthetic Pathways

Following the structural determination of gibberellic acid (GA_3_), experiments to determine its biosynthetic origin in *G. fujikuroi* began in the late 1950s. Incorporation of ^14^C-labelled substrates, including acetate and mevalonate (MVA), into GA_3_ in fungal cultures followed by degradation confirmed its diterpenoid nature (Birch and others 1958). Later Cross and others (1964) demonstrated that the tetracyclic diterpene hydrocarbon, (-)-kaurene, now more commonly referred to as *ent*-kaurene, was incorporated into GA_3_, establishing it as an intermediate. At about this time, Jan Graebe, who was a graduate student working with Bernard Phinney and Charles West at UCLA, attempted to prepare cell-free preparations from the fungal mycelia, but achieving no success turned instead to the endosperm-nucellus of the California wild cucumber (*Marah macrocarpus*, formerly *Echinocystis macrocarpa*), which Phinney and others (1957) had shown to be a rich source of gibberellin-like substances. Jan Graebe’s endosperm system was extremely active and he could demonstrate conversion of MVA into *ent*-kaurene and *ent*-kaurenol (Graebe and others 1965). Later, on establishing his own laboratory at the University of Göttingen, Germany, Jan Graebe continued to work on GA biosynthesis in endosperm of another member of the Cucurbitaceae, pumpkin (*Cucurbita* species), with considerable success (see below). Following this first demonstration of *ent*-kaurene synthesis, cell-free systems from a number of other plant sources, mainly developing seeds, were shown to convert MVA into *ent*-kaurene, but the *Marah* and *Cucurbita* systems, in contrast to the others, produced *ent*-kaurene as the major product in high yield (reviewed in Hedden and others 1978).

**Fig. 1:**
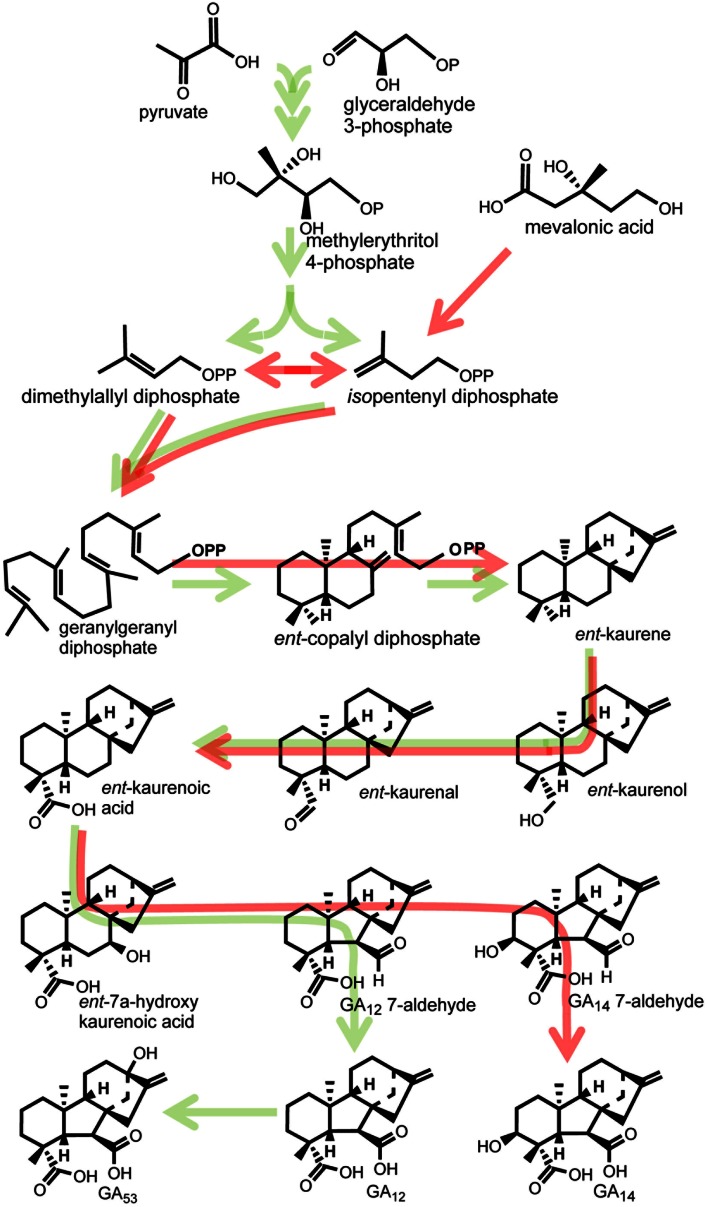
Early and intermediate steps of GA biosynthesis in higher plants (*green arrows*) and the fungus *Fusarium fujikuroi* (*red arrows*). In plants, *ent*-kaurene is synthesised in plastids, predominately via the methylerythritol phosphate pathway, while in fungi, it is biosynthesised from mevalonic acid. Conversion of *ent*-kaurene to GA_12_ and GA_53_ (plants) and GA_14_ (fungi) is catalysed by membrane-associated cytochrome P450 monooxygenases. *Arrows* running through structures indicate multiple steps catalysed by single enzymes

**Fig. 2:**
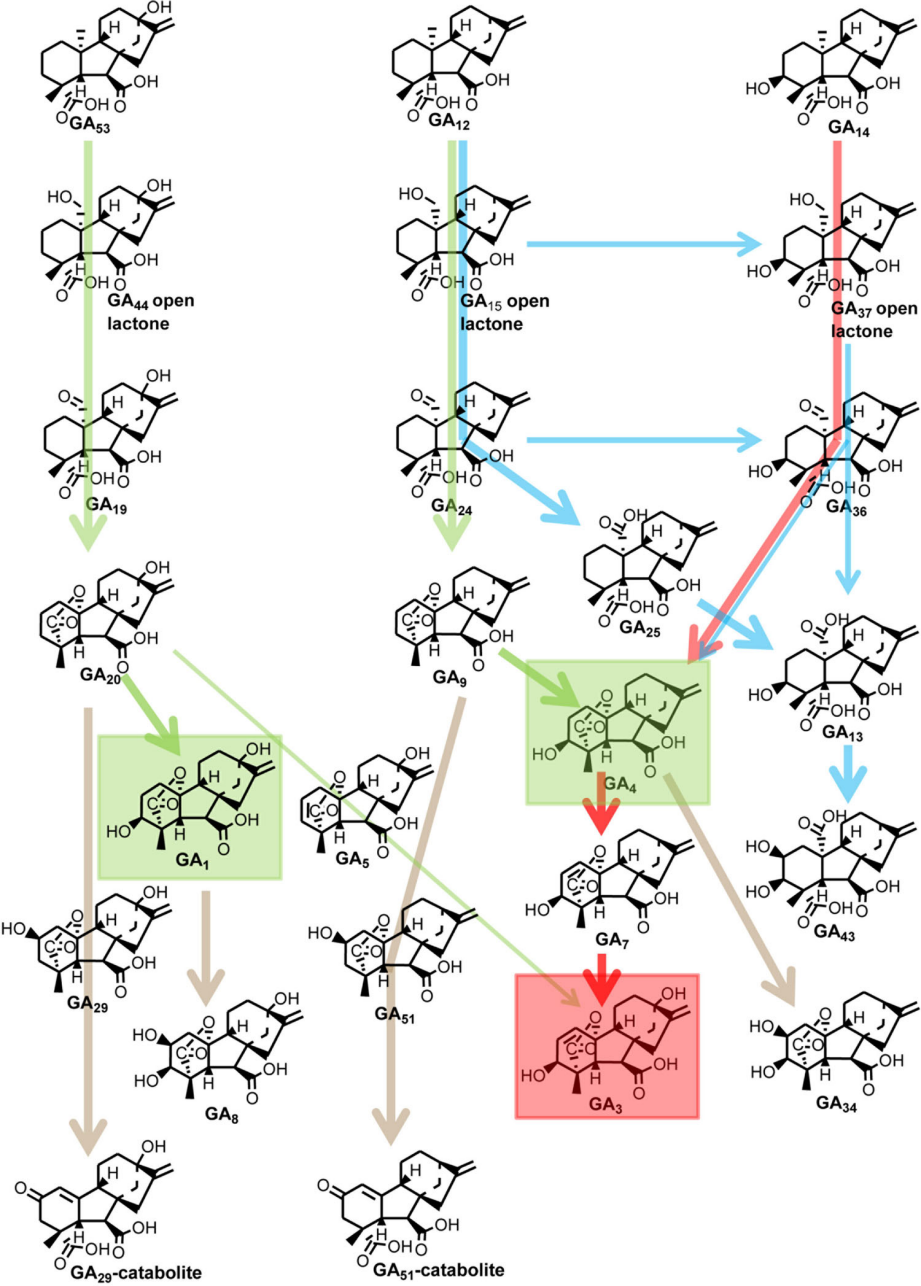
Late steps of GA biosynthesis in vegetative plant tissues (*green* and *brown arrows*), pumpkin endosperm (*blue arrows*) and the fungus *Fusarium fujikuroi* (*red arrows*). The main bioactive GAs in plants, GA_1_ and GA_4_, are boxed in *green*, while the product of the fungal pathway, GA_3_, which is also active and produced as a minor product in some plants, is boxed in *red*. *Brown arrows* indicate inactivation of C_19_-GAs by 2β-hydroxylation and further C-2 oxidation to catabolites (shown for GA_29_ and GA_51_, but can also occur for GA_8_ and GA_34_). The reactions are catalysed by soluble 2-oxoglutarate-dependent dioxygenases in plants and cytochrome P450 monooxygenases in the fungus, except for the fungal desaturase that converts GA_4_ to GA_7_, which is a 2-oxoglutarate-dependent dioxygenase. *Arrows* running through structures indicate multiple steps catalysed by single enzymes

The main pathways for GA biosynthesis in *G. fujikuroi*, pumpkin endosperm and vegetative organs of higher plants are shown in Figs. 1 and 2. The pathways in the fungus and plants differ in the order of the 3β-hydroxylation and 13-hydroxylation steps, the former occurring early in the fungus (Fig. 1), whereas it is the last step in plants (Fig. 2). In contrast, 13-hydroxylation commonly occurs before loss of C-20 in plants and is the last step in GA_3_ biosynthesis in *G. fujikuroi.* Work on GA biosynthesis in *G. fujikuroi* and higher plants, mainly in cell-free systems from developing seeds, continued in parallel in the 1960s and 1970s, resulting in the main features of the pathways being established. Many of the initial experiments were conducted in Charles West’s laboratory at UCLA. A cell-free system from *G. fujikuroi* mycelia established *trans*-geranylgeranyl diphosphate as a precursor of *ent*-kaurene, to which it is converted in two steps via *ent*-copalyl diphosphate (Fall and West 1971; Shechter and West 1969). The sequence of steps from *ent*-kaurene to *ent*-kaurenoic acid and then to *ent*-7α-hydroxykaurenoic acid was shown in the *Marah* and pumpkin cell-free systems (Dennis and West 1967; Graebe 1972; Lew and West 1971). These steps were subsequently confirmed in a number of other cell-free and intact systems (reviewed in Hedden and others 1978). The next intermediate, GA_12_-aldehyde, the first with the *ent*-gibberellane carbon skeleton, was shown to be formed from MVA in the pumpkin cell-free system, which also produced GA_12_ (Graebe and others 1972). No intermediates beyond GA_12_ were obtained until Mn^2+^, which was included to enhance *ent*-kaurene formation, was omitted from the cell-free system. In the absence of Mn^2+^, GA_12_ was converted to a number of products, included GA_43_, which is a major endogenous GA in pumpkin endosperm, and to the C_19_-GA, GA_4_ (Graebe and Hedden 1974; Graebe and others 1974a, b). Refeeding these products established the pathway shown in Fig. 2 (blue arrows). The reason for the inhibition of these later reactions by Mn^2+^ became apparent once the nature of the enzymes catalysing these steps was established (see below).

In the 1970s, considerable progress was made in determining the GA-biosynthetic pathways in *G. fujikuroi.* This included particularly work conducted in MacMillan’s laboratory in Bristol using liquid cultures of a GA-deficient mutant, B1-41a, provided by Phinney (Bearder and others 1975). B1-41a contains a lesion in *ent*-kaurene oxidase and allowed unlabelled substrates to be used without dilution by endogenous metabolites. These experiments and those in James Hanson’s laboratory at the University of Sussex, UK, showed that 3β-hydroxylation occurred on GA_12_-aldehyde and that GA_14_, but not GA_12,_ was on the pathway to GA_3_ (Bearder and others 1975; Evans and Hanson 1975). Hanson and colleagues determined the stereochemistry of many of the steps, showing, for example, that the 1α, 2α-H atoms are lost in the dehydrogenation of GA_4_ to form GA_7_ (Evans and others 1970). However, it was not possible to determine the immediate precursor from which C-20 was lost in the formation of C_19_-GAs since the potential C_20_ intermediates in the oxidation of C-20, GA_37_ and GA_36_ did not accumulate and were not metabolised by the fungal cultures. In contrast, since the oxidised C_20_ precursors accumulate in plants and are readily metabolised, it was possible to show using cell-free systems from pumpkin endosperm and pea seeds that C-20 was lost from the aldehyde (Graebe and others 1980; Kamiya and Graebe 1983). This confirmed earlier suggestions by Hanson and White (1969) and Durley and others (1974). The difference between the fungus and higher plants could be later explained by the nature of the GA 20-oxidase enzymes that catalyse the sequential oxidation of C-20, the fungus utilising a cytochrome P450 monooxygenase for these reactions as opposed to a 2-oxoglutarate-dependent dioxygenase in plants (Hedden and others 2002). Although it has not been demonstrated, it is likely that the oxidised C-20 intermediates in the fungus are not released by the enzyme during this multi-step reaction.

The biosynthetic pathways in plants were also being studied in intact organs at this time. Developing seeds of legumes are particularly rich in GAs and have been used both as intact and cell-free systems to study the later stages of biosynthesis. Notably, experiments by Sponsel and MacMillan (1977) in which substrates were injected into cotyledons of immature pea seeds provided evidence of two parallel pathways leading to 13-hydroxylated and 13-deoxy GAs, respectively, with 13-hydroxylation occurring early in the pathway. This system also demonstrated high levels of 2β-hydroxylation, particularly in the testa, with the 2β-hydroxylated C_19_-GA products being further oxidised on C-2 to form the GA catabolites (Sponsel 1983). Because 2β-hydroxy GAs have low biological activity, their formation was recognised as an inactivation process, which is important in the regulation of bioactive GA concentrations. It does not occur in *G. fujikuroi*, in which oxidation at C-2 occurs on the α face. Other inactivation mechanisms known to occur in plants are conjugation, primarily with glucose (Schneider and Schliemann 1994) and the recently described epoxidation of the C-16,17 double bond (Zhu and others 2006). The epoxides are hydrated to the 16,17-dihydrodiols, which have been known for some years to be endogenous GA metabolites (for example, Hedden and others 1993).

Several of the biosynthetic steps were of particular interest from a mechanistic standpoint. The formation of *ent*-kaurene from *ent*-copalyl diphosphate involves a complex rearrangement proposed to arise from a carbonium ion formed by heterolytic cleavage of the diphosphate (evidence reviewed in MacMillan and Beale 1999). Contraction of ring C from six to five carbons in the formation of GA_12_-aldehyde from *ent*-7α-hydroxykaurenoic acid occurs with extrusion of C-7. It was proposed (Evans and others 1970) and subsequently confirmed (Castellaro and others 1990; Graebe 1980; Graebe and others 1975) that ring contraction is initiated by stereospecific removal of the C-6β H atom. In pumpkin endosperm and the fungus *G. fujikuroi*, a by-product, *ent*-6α, 7α-dihydroxykaurenoic acid, accompanies GA_12_-aldehyde formation. Thus, the intermediate formed after the removal of the 6β-H, assumed to be a free radical, undergoes either rearrangement and further loss of H^**∙**^ to give GA_12_-aldehyde or recombines with HO^∙^ to form the dihydroxykaurenoic acid. This latter product is further oxidised, but is not converted to GAs. Other by-products of GA biosynthesis are formed in pumpkin endosperm and *G. fujikuroi*, in which *ent*-kaurenoic acid is converted to *ent*-kaur-6,16-dienoic acid (and then to the kaurenolides), by stereospecific removal of the 6α, 7α-H atoms (Beale and others 1982; Castellaro and others 1990; Hedden and Graebe 1981). It has been shown for the fungus that all of these by-products are produced by the highly multifunctional enzyme that converts *ent*-kaurenoic acid to GA_14_ (Rojas and others 2001). The equivalent enzyme, *ent*-kaurenoic acid oxidase, in pumpkin endosperm can be assumed to have similar catalytic properties, although it lacks 3β-hydroxylase activity, forming GA_12_ rather than GA_14_. However, there is no evidence for these by-products being formed in vegetative plant tissues, which presumably possess *ent*-kaurenoic acid oxidases with tighter specificity. The mechanism for the loss of C-20 from the aldehyde is still unclear. Bearder and others (1976) showed that in the fungus both oxygen atoms in the γ-lactone of C_19_-GAs were derived from the carboxylic acid on C-4 (C-19). Later Yuji Kamiya demonstrated in a cell-free system from developing pea cotyledons that C-20 is lost from the aldehyde as CO_2_ (Kamiya and others 1986). This would require two oxidation steps, although no intermediate between the aldehyde and C_19_ product has been identified. The GA 20-oxidase (GA20ox) enzyme responsible for removing C-20 also catalyses the oxidation of C-20 from a methyl to the aldehyde via an alcohol. More recently, on the basis of experiments with a recombinant GA20ox from *Arabidopsis*, it was proposed that an initially formed free radical on C-20 decomposes by an unknown oxidative mechanism to produce a C-10 radical, which captures the C-4 carboxyl group (Ward and others 2002).

### The Enzymes

The properties of the diterpene cyclases that convert GGDP to *ent*-copalyl diphosphate and then to *ent*-kaurene were first studied in West’s laboratory at UCLA. The activities were originally named *ent*-kaurene synthetase A and B (erroneously as they do not require ATP), but the names *ent*-copalyl diphosphate synthase (CPS) and *ent*-kaurene synthase (KS) were proposed by MacMillan (1997) and have been universally adopted. In the fungus, the two activities reside on a single polypeptide, which was purified by Fall and West in 1971. The activities were partially purified from *M. macrocarpus* and could also not be separated (Frost and West 1977), although subsequent work showed them to be separate enzymes, which may act in association (Duncan and West 1981). Early indications that *ent*-kaurene synthesis occurred in plastids (for example, Simcox and others 1975) were later confirmed by Aach and others (1995), who showed conclusively that GGDP was converted to *ent*-kaurene in plastids from pea shoot tips and pumpkin endosperm. Furthermore, following the cloning of their cDNAs (see below), both CPS and KS were found to contain transit sequences for plastid targeting. It is notable that despite the many demonstrations of *ent*-kaurene synthesis from MVA in cell-free systems, *ent*-kaurene was later shown to be produced mainly from pyruvate and glyceraldehyde phosphate via the methylerythritol phosphate (MEP) pathway in plants (Fig. 1; Kasahara and others 2002).

Work with cell-free preparations from *Marah*, pumpkin, pea and *Gibberella* showed that the oxidative activities for the conversion of *ent*-kaurene to GA_12_-aldehyde were present in microsomes and were stimulated by NADPH. West and colleagues demonstrated that the enzymes catalysing the conversion of *ent*-kaurene to *ent*-7α-hydroxykaurenoic acid had the properties of cytochrome P450-dependent monooxygenases (Hasson and West 1976a; b; Murphy and West 1969). In the pumpkin cell-free system, GA_12_-aldehyde is oxidised to GA_12_ by both microsomal and soluble enzymes (reviewed in Hedden 1983), whereas a microsomal preparation from pea cotyledons converted GA_12_-aldehyde to GA_12_ and thence to GA_53_ by 13-hydroxylation (Ropers and others 1978). Thus, it was demonstrated that in higher plants, the middle section of the pathway from *ent*-kaurene to GA_12_ and GA_53_ was catalysed by monooxygenases. After the cloning of cDNAs encoding these enzymes (see below), it was found that just two enzymes, *ent*-kaurene oxidase (KO) and *ent*-kaurenoic acid oxidase (KAO), were required for GA_12_ formation from *ent*-kaurene, with a third enzyme responsible for 13-hydroxylation. The demonstration in Graebe’s laboratory that these enzymes were present in the endoplasmic reticulum (Graebe 1980) was later confirmed using GFP fusions by Helliwell and others (2001a, b), who showed that KO was also present in the plastid envelope. The fungal cell-free system being investigated in West’s laboratory was capable of forming GA_14_, but no activity could be obtained for the further steps (West 1973). There is still no explanation for this conundrum. After the early 1980s, there was a hiatus in research on fungal GA biosynthesis, but the topic was reactivated by Bettina Tudzynski and her collaborators in the late 1990s through the identification and characterisation of the biosynthetic genes, which are present as a cluster. Through targeted gene knock-out and expression of individual genes in a mutant strain lacking the gene cluster, they could demonstrate the function of each of the seven enzymes responsible for GA_3_ biosynthesis (Linnemannstöns and others 1999; Tudzynski and others 2003). With the exception of a 2-oxoglutarate-dependent dioxygenase that converts GA_4_ to GA_7_ (Bhattacharya and others 2012), the steps from *ent*-kaurene, including the 20-oxidation of GA_14_ to GA_4_, are catalysed by cytochrome P450 monooxygenases.

When conversion of GA_12_-aldehyde and GA_12_ to endogenous GAs was achieved with the pumpkin endosperm system, there was considerable interest in discovering the nature of the enzymes, which were found to be soluble and therefore different from the monooxygenases responsible for the earlier steps (Graebe and Hedden 1974). Experiments with this system and with others, such as those from *Phaseolus* seeds (Patterson and others 1975), indicated that the enzymes required Fe^2+^, which could be removed by Fe chelators such as EDTA. This explained the inhibition by Mn^2+^ and other heavy metal ions which could displace Fe at the enzyme active site. Enzyme activity was lost after gel filtration, indicating the requirement for a small molecule cofactor. The demonstration that activity could be restored by 2-oxoglutaric acid and stimulated by ascorbic acid established the enzymes to be 2-oxoglutarate-dependent dioxygenases (ODDs) (Hedden and Graebe 1982). Four potential ODD activities were present in the pumpkin system: 20-oxidation, 3β-hydroxylation and 2β-hydroxylation, which are universal in higher plants, and the 7-oxidation of GA_12_-aldehyde to GA_12_. This last enzyme appears to have a restricted distribution, being so far identified in members of the Cucurbitaceae (Pimenta Lange and others 2013). After the identification of the enzymes, the next step was to purify them, and this was undertaken in several laboratories, particularly in order to facilitate their cloning (Griggs and others 1991; Kwak and others 1988; Lange and others 1994b; Smith and MacMillan 1984). In fact, cloning was enabled both by enzyme purification and the use of mutants.

### Mutants and Genes

The importance of GA-deficient mutants of pea and maize in establishing GAs as plant hormones has already been described. These and mutants in other species, most notably *Arabidopsis*, were to prove extremely valuable for studies on GA biosynthesis and in identifying transcripts and genes encoding the enzymes. Bernard Phinney at UCLA and Ian Murfet in Hobart, Tasmania assembled a series of single gene mutants of maize and pea, respectively, for which, through a combination of substrate feeding and product identification by GC–MS, the sites of the lesions in the biosynthetic pathway were identified. For example, the *dwarf*-*1* and *le* mutants of maize and pea, respectively, were shown to be defective in the 3β-hydroxylation of GA_20_ to GA_1_ (Ingram and others 1984; Spray and others 1984). There was particular excitement in defining the *le* lesion because it was responsible for one of the traits (difference in stem height) used in Mendel’s classic experiments on the nature of inheritance. Later, the cloning of the *LE cDNA* allowed the amino acid substitution, causing impairment of enzyme function in the *le* mutant to be defined (Lester and others 1997; Martin and others 1997). The first characterisation of a GA-biosynthetic mutation was reported for maize, in which it was demonstrated using cell-free systems from shoots that the *dwarf*-*5* mutant was defective in KS activity, producing *ent*-isokaurene rather than *ent*-kaurene (Hedden and Phinney 1979). Also in maize, Phinney and Spray (1982) demonstrated in bioassays with *dwarf*-*1* that GA_1_, but none of its precursors, possessed biological activity, so confirming the structural requirements for activity, which were later substantiated when the GA receptor was identified (see below).

In 1980, Maartin Koornneef at Wageningen, The Netherlands, produced a number of GA-sensitive mutants in *Arabidopsis*, naming them *ga1* to *ga5* on the basis of epistasis (Koornneef and van der Veen 1980). The *ga1*, *ga2* and *ga3* mutants were extreme dwarfs, and were sterile with non-germinating seeds, whereas the *ga4* and *ga5* phenotypes were much less severe. Analysis of the GAs in *ga4* and *ga5* by Talon and others (1990b) indicated that they were defective in 3β-hydroxylation and 20-oxidation, respectively. Redundancy for the GA3ox and GA20ox enzymes catalysing these reactions was later to explain the relatively mild phenotype, whereas the *GA1*, *GA*2 and *GA3* genes are present as single copies. The *ga1*-*3* mutant, which was produced by neutron bombardment and contained a large deletion, was utilised in the first cloning of a GA-biosynthetic gene using genomic subtraction (Sun and others 1992). *GA1* could then be shown by expression in *E. coli* to encode CPS (Sun and Kamiya 1994). Soon after, the *Anther ear1* (*An1*) gene of maize, predicted to encode CPS, was cloned by transposon tagging (Bensen and others 1995). Both *GA1* and *AN1* contained chloroplast-targeting leader sequences.

The cloning of the *ArabidopsisCPS* was quickly followed by the identification of cDNAs for the other biosynthetic enzymes. Theo Lange working with Jan Graebe and Peter Hedden purified a GA20ox from pumpkin endosperm and obtained partial sequences (Lange 1994), allowing the production of peptide antibodies that were used to isolate the cDNA from an expression library (Lange and others 1994a). The identity of the clone was confirmed by functional expression in *E. coli*. The nucleotide sequence of the pumpkin clone allowed the isolation of three *GA20ox* cDNAs from *Arabidopsis* through PCR by Andy Phillips at Long Ashton Research Station, UK (Phillips and others 1995). The three GA20ox enzymes were functionally similar, oxidising GA_12_ to the C_19_-GA, GA_9_, in contrast to the pumpkin GA20ox that produced the tricarboxylic acid GA_25_ as the major product. Expression of the genes showed different tissue specificity and was down-regulated by application of GA, confirming feedback regulation (see later). A similar strategy was used in Jan Zeevaart’s laboratory to clone one of the *ArabidopsisGA20ox* cDNAs, which they showed to correspond to *GA5* (Xu and others 1995). T-DNA tagging enabled Chiang and others (1995) to clone the *ArabidopsisGA4* gene, which was later confirmed to encode a GA3ox by expression in *E. coli* (Williams and others 1998). Shinjiro Yamaguchi, working with Yuji Kamiya at the RIKEN in Wako, Japan, cloned KS from pumpkin cotyledons after purifying the enzyme (Yamaguchi and others 1996), allowing him to isolate the homologous cDNA from *Arabidopsis* and, through mutant complementation, demonstrate its identity with *GA2* (Yamaguchi and others 1998)*. GA3* was cloned by Helliwell and others (1998) at the CSIRO laboratory in Canberra, Australia, by map-based cloning and random sequencing. The same group cloned *KAO* from barley, where it is defined by the *grd5* mutation, and then from *Arabidopsis*, which contains two fully redundant copies (Helliwell and others 2001a). They demonstrated that the enzymes carry out the three-step conversion of *ent*-kaurenoic acid to GA_12_ by heterologous expression in yeast.

The availability of these genes provided a means to modify GA content through ectopic expression in transgenic plants. Such studies showed that in *Arabidopsis*, GA biosynthesis is limited particularly by GA20ox activity (Coles and others 1999; Fleet and others 2003; Huang and others 1998). The potential benefits of modifying GA metabolism in crop species were a powerful driver for such experiments, particularly with the aim of reducing GA content to control growth. Chemical growth retardants had been available since 1949 (Mitchell and others 1949), with notable early examples being 2′-isopropyl-4′- (trimethylammonium chloride) -5′-methylphenyl piperidine-1-carboxylate (AMO-1618; Wirwille and Mitchell 1950) and chlormequat chloride (CCC; Tolbert 1960), the latter still in use primarily as an anti-lodging agent. As growth inhibition by these chemicals could be reversed by application of GAs, they were thought to function as anti-gibberellins, and they were found to inhibit GA biosynthesis in the fungus (Kende and others 1963). Subsequently, AMO-1618 and other quaternary ammonium-type inhibitors were shown to inhibit *ent*-kaurene synthesis (Dennis and others 1965). Further growth retardants acting on different stages of the biosynthetic pathway have been developed, with KS, KO and GA3ox, the principal sites of action (reviewed by Rademacher 2000). As an alternative to growth retardants, expression of GA deactivating genes, such as *GA2ox* (encoding 2β-hydroxylases), was an attractive option. To isolate *GA2ox* clones, Steve Thomas, working with Peter Hedden and Andy Phillips, returned to the material from which GAs were first identified, immature *P. coccineus* seeds, a known rich source of 2β-hydroxylase activity (Durley and others 1971). The simple successful strategy involved screening a cDNA expression library for clones that released ^3^H from [1,2-^3^H_2_]GA_9_ (Thomas and others 1999). The bean enzyme and three GA2ox enzymes identified by homology from *Arabidopsis* accepted C_19_-GA substrates, oxidising them to 2β-hydroxy products, with some also producing GA catabolites. *GA2ox* cDNAs were subsequently cloned from immature pea cotyledons by Dave Martin working with William Proebsting in Corvallis, Oregon, and Diane Lester in James Reid’s group in Hobart (Lester and others 1999; Martin and others 1999). Later, a new class of GA2ox which hydroxylates C_20_-GAs was identified in *Arabidopsis* by activation tagging (Schomburg and others 2003). Both classes of GA2ox are ubiquitous in higher plants and have important roles in regulating GA content. Their overexpression has proved to be a very effective method for producing dwarfism (Phillips 2004).

The recent cloning of GA_12_ 13-hydroxylases from rice (Magome and others 2013) means that genes have now been identified for all the enzymes in the pathway. The two rice cDNAs encode cytochrome P450 monooxygenases that are closely related to the inactivating 16, 17-epoxidase (EUI). Indeed, Magome and others (2013) suggested that 13-hydroxylation may be a form of mild deactivation because overexpression of these cDNAs caused reduced growth. This is an interesting and unexpected conclusion since in most plant species, *Arabidopsis* being a notable exception, the 13-hydroxylation pathway predominates.

The increasing number of plant genome sequences now available has simplified the identification of GA-biosynthetic genes. However, the genes are often incorrectly annotated, and in only a few cases, their functions are demonstrated biochemically, being assigned on the basis of sequence homology. The focus of research on GA metabolism has now moved to its regulation by developmental and environmental factors and the determination of the underlying mechanisms. This topic has been covered in a recent review (Hedden and Thomas 2012).

## Gibberellin Action

**Fig. 3:**
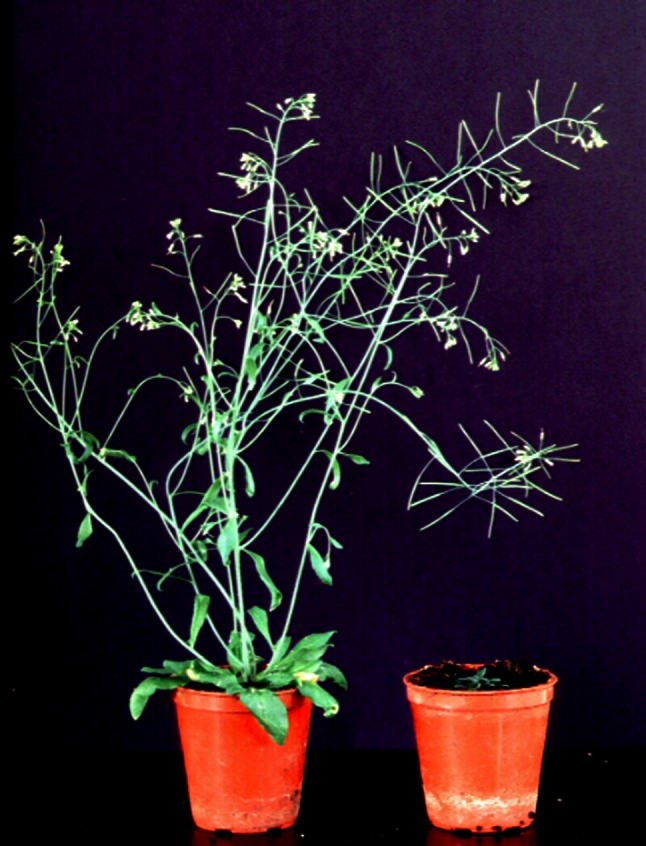
Physiological action of GA as illustrated by comparison of the Landsberg *erecta* *Arabidopsis* plant with a GA-deficient mutant (ga1-3). In the absence of a GA response stem elongation, leaf enlargement, floral development, seed set and fruit development do not occur

Investigations into the physiological responses of higher plants to GA were advanced even before the active compounds had been isolated and structurally characterised. The early work was reviewed by Stowe and Yamaki (1957), who listed the numerous effects of GAs on plant development. Some of these are illustrated in Fig. 3, which compares a wild-type *Arabidopsis* plant with a GA-deficient mutant. With remarkable foresight, they noted that “there is little doubt that the gibberellins must correspond in their action to naturally-occurring compounds in higher plants” and suggested that GA acts by removing a limitation to growth. Promotion of elongation in young (still growing) stems is one of the most obvious effects of GA and it occurs without a change in the number of nodes. Internode growth is promoted through enhanced cell elongation, shown later to be due to relaxation of the cell wall rather than increased cell turgor (Cosgrove and Sovonick-Dunford 1989). However, GAs also promote cell division in some circumstances, notably in the induction of bolting in rosette species (Sachs 1965). Stowe and Yamaki noted that GA promotes leaf expansion, but inhibits root growth, from which they concluded that GA changes the root–shoot ratio. It is now known that GA action is essential for root elongation, but high GA concentrations are inhibitory and in most cases roots contain close to saturating GA levels (Tanimoto 2012). Another notable action of GA is the promotion of seed germination: of particular note was the observation that GA substituted for the light requirement for germination of photoblastic seeds, whereas it reversed the light inhibition of stem elongation. These contrasting effects could be later explained by the opposite responses of GA metabolism to red light in these tissues (reviewed by Kamiya and Garcia-Martinez 1999). The effect of GAs on flowering is complex and can be promotive, inhibitory or neutral depending on the species (Pharis and King 1985; Zeevaart 1976). Some long-day plants growing under non-inductive conditions can be induced to bolt and flower by GA application, while others will bolt without flowering. The ability of GA to substitute for long-days prompted speculation that it was the long sought-after leaf-derived signal, florigen, and, although this is now generally recognised to be flowering locus T (FT) or related peptides, there is no doubt that GA can act as a mobile inductive signal, if not the major one (King 2012). Until the discovery of GAs, elongation growth was thought to be regulated exclusively by auxin, and many of the early experiments tested the hypothesis that GA acted by stimulating auxin levels (reviewed in Paleg 1965). However, the reverse scenario is now known to occur with auxin promoting stem elongation by increasing GA biosynthesis (Ross and others 2001), although in a recent report, it was shown that GA is required for auxin transport (Willige and others 2011).

The molecular mechanisms of GA action has in recent years become intensively researched, but for practical reasons, much of the early research on GA function was conducted with germinating cereal grain. Germination in cereals is associated with the production and secretion of hydrolytic enzymes, including α-amylase, in the aleurone layer for the breakdown of macromolecules in the endosperm as a source of nutrient for the growing embryo. Research on this topic was stimulated by the importance of the process for malt production in brewing. In 1940, Takeshi Hayashi, working at the Imperial Agricultural Station, Hongo, Tokyo showed that barley grain germination and amylase activity were stimulated by GA (Hayashi 1940). The topic was reactivated in 1960 when Leslie Paleg, at the Waite Institute in Adelaide, Australia and Harugoro Yomo, working at the Takara Shuzo Company in Kyoto, Japan, reported independently that GA stimulated amylase production in embryo-less barley grain (Paleg 1960a; Yomo 1960). Margaret Radley (1959) had shown earlier that barley grain contained GA-like substances which increased during germination, prompting the suggestion that the embryo was the source of GA that stimulated amylase production in the endosperm (Paleg 1960b). This proposal has been substantiated many times since (reviewed in Bethke and others 1997). It was later shown by Chrispeels and Varner (1967) that the source of α-amylase was the aleurone, a layer of living cells surrounding the dead starchy endosperm.

The cereal aleurone proved an ideal experimental system to study GA action, since it was dependent on an external source of GA and gave a well-defined biochemical response. It could be easily isolated to produce a uniform population of cells and was amenable to the production of protoplasts which retain their GA response (with some changes), allowing experiments on membrane properties, such as patch clamping, unencumbered by the cell wall. Gibberellin was shown to promote α-amylase mRNA production in the barley aleurone (Higgins and others 1976), but the response occurs relatively late and is preceded by increases in cytosolic free Ca^2+^, changes in cytosolic pH, and in the concentrations of calmodulin and cyclic GMP (reviewed in Bethke and others 1997). The role of these factors in the GA response is still not well understood. It has, however, been established that GA promotes expression of a MYB transcription factor (termed GAMYB), which binds to the promoters of α-amylase genes and activates their expression (Gubler and others 1995). *GAMYB* mRNA production following GA treatment is not affected by the translation inhibitor cycloheximide, indicating that *GAMYB* may be a primary response gene. GA was shown also to promote programmed cell death of aleurone cells (Bethke and others 1999), a process that also occurs in the tapetum via a GA-regulated mechanism involving GAMYB (reviewed in Plackett and others 2011).

A number of lines of evidence indicated that the GA receptor in aleurone cells was present on the plasma membrane. Although membrane-impermeable GA induced α-amylase production in oat aleurone protoplasts (Hooley and others 1991), GA injected into barley aleurone protoplasts was ineffective (Gilroy and Jones 1994). Furthermore, experiments with an agonist and inhibitor of heterotrimeric G proteins suggested their involvement in the response of the oat aleurone to GA (Jones and others 1998). However, a membrane GA receptor has not been identified, and the discovery of a soluble, nuclear-localised GA receptor (GID1) in rice (Ueguchi-Tanaka and others 2005) has placed some doubt on its existence, particularly with the recent report that *GID1* was the only GA receptor in rice (Yano and others 2015). Indeed there is some debate as to whether plants actually contain G-protein coupled receptors (Taddese and others 2014). Nevertheless, the demonstration that the rice GA-insensitive *dwarf1* mutant is defective in the Gα subunit of a heterotrimeric G protein suggests that these proteins may play some role in GA signalling (Ueguchi-Tanaka and others 2000).

**Fig. 4:**
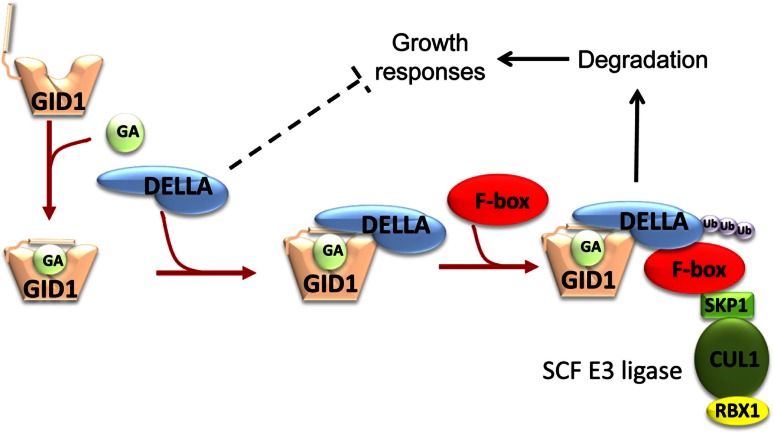
Representation of GA perception and signal transduction. Binding of a bioactive GA results in a conformational change in the GID1 receptor that promotes interaction with DELLA proteins. Recruitment of an F-box protein initiates ubiquitination of DELLA by an SCF E3 ubiquitin ligase targeting the DELLA for proteasomal degradation. Loss of DELLA relieves growth repression and suppresses other DELLA-mediated responses

The mechanism by which GAs promote growth, summarised in Fig. 4, has been formulated over the last 20 years, with particular progress following the discovery of the GID1 receptor in 2005. The basic concept that GAs act by suppressing a growth inhibitor was proposed from studies with GA-insensitive mutants (Harberd and others 1998). The characteristics of such mutants had been known for many years. In 1970, Margaret Radley showed that the Japanese dwarf wheat cultivar Norin-10 and related dwarf lines did not respond to applied GA, unlike tall lines, and that they accumulated much higher levels of GA-like substances than the tall cultivars (Radley 1970). She suggested that in these lines, a “block to the utilisation of GA causes an accumulation of the hormone”. This proposal proved correct, although the link between GA action and metabolism was not as direct as Radley may have envisaged. Norin-10 is the source of the *Reduced height* (*Rht*) genes that were introduced by Norman Borlaug into high yielding wheat varieties in the Green Revolution to stabilise the stem and increase harvest index (Hedden 2003). The two homoeologous semi-dwarfing genes present in Norin-10, *Rht1* (renamed *RhtB1b* to indicate its genome location and allele) and *Rht2* (*RhtD1b*), are still used widely in modern wheat cultivars. Appleford and Lenton (1991) showed that near isogenic lines containing *Rht*-*B1b* or the more severe *Rht*-*B1c* (*Rht3*) dwarfing allele accumulate C_19_-GAs, but have reduced levels of C_20_-GAs compared with the tall (*Rht*-*B1a*) line. Similar results had been obtained for the GA-insensitive *dwarf*-*8* mutant of maize (Fujioka and others 1988) and *GA*-*insensitive* (*gai*) (Talon and others 1990a), an *Arabidopsis* deletion mutant obtained by Koornneef and others (1985). In contrast, Potts and others (1985) reported that slender pea mutants containing the *la cry*^*s*^ gene combination grew independently of GA status and possessed abnormally low levels of GA-like substances. Similarly, *slender*, an overgrowth mutant of barley with a constitutive GA response was shown to contain lower levels of C_19_-GAs, but elevated C_20_-GA levels relative to its wild type (Croker and others 1990). On the basis of these observations and the ability to normalise GA precursor levels in the maize *dwarf1* (3β-hydroxylase) mutant by treating with GA, Hedden and Croker (1992) proposed that GA action resulted in reduced GA20ox activity, that is, GA20ox was under feedback regulation. When *GA20ox* cDNAs were cloned from *Arabidopsis*, transcript abundance for these genes was shown to be regulated by GA (Phillips and others 1995). The demonstration by Cowling and others (1998) that the transcript level for *GA4*, which encodes a GA3ox enzyme (AtGA3ox1), was similarly regulated by GA signalling extended the number of genes under feedback control. Subsequently, it was reported that some *GA2ox* genes are up-regulated by GA (Thomas and others 1999), whereas the *GID1* receptor genes are down-regulated (Griffiths and others 2006), indicating the existence of a complex system of homeostatic regulation in GA signalling.

A breakthrough in GA signalling was achieved with the cloning from *Arabidopsis* of the genes responsible for GA-insensitivity. Nicholas Harberd and colleagues at the John Innes Centre, Norwich, UK, cloned *GAI* and a related gene *GRS* and demonstrated that the gai mutant contained a 17-amino acid deletion in the N-terminal region (Peng and others 1997). On the basis of genetic evidence, they proposed that GAI, which had the characteristics of a transcriptional co-activator, is a growth repressor, that the repression is relieved by GA signalling, and that the gai mutant form is resistant to GA, that is, gai is a gain-of-function mutation. Tai-ping Sun and colleagues at Duke University, USA, substantiated this scenario when they characterised a loss of function mutation that partially rescued the semi-dwarf phenotype of the GA-deficient mutant *ga1*-*3* (Silverstone and others 1997). They showed that the gene, called *REPRESSOR of ga1*-*3* (*RGA*), was identical to *GRS* and that the encoded protein, which was 82 % similar to GAI, was degraded by GA signalling (Silverstone and others 1998). Furthermore, a mutant form of RGA with the same deletion as in gai was resistant to GA-induced degradation. Thus, the N-terminal region is required for degradation in the presence of GA, but not for growth repression. GAI and RGA belong to a plant-specific family of transcriptional regulators, named GRAS after its first three members, GAI, RGA and SCARECROW (Pysh and others 1999), but GAI and RGA form a subgroup of GRAS proteins with conserved DELLA and VHYNP motifs at the N-terminus not present in SCARECROW and related proteins. These motifs are essential for GA-regulated degradation of this subgroup (Dill and others 2001), known as DELLA proteins (Wen and Chang 2002). After the identification of *GAI* and *RGA*, other DELLA genes were cloned: *Arabidopsis* was found to contain three further DELLA proteins, RGA-like1, -2 and -3 (Hussain and Peng 2003), while, of particular significance, the Harberd group showed that wheat *Rht* and maize *Dwarf*-*8* encode DELLA proteins and that the gain-of-function mutations that produce GA-insensitivity are due to disruption to the N-terminus (Peng and others 1999). As the *Rht*-*B1b* and *Rht*-*D1b* mutations create stop codons in the DELLA region, it was assumed, although it has still not been demonstrated, that re-initiation of translation produces a truncated product lacking the DELLA motif. As for wheat and maize, barley and rice contain a single DELLA protein, SLN1 and SLR1, respectively (Chandler and others 2002; Ikeda and others 2001). Strikingly, missense mutations in their N-terminus produce dwarfism (gain of function), while loss of function mutations result in an overgrowth (slender) phenotype.

Further progress was made in 2003, when the *ArabidopsisSLY1* and rice *GID2* genes were cloned and shown to encode the F-box components of SCF ubiquitin ligases (McGinnis and others 2003; Sasaki and others 2003). Mutations in these genes caused an accumulation of DELLA protein and GA-insensitive dwarfism suggesting that DELLA degradation involved ubiquitination, which targeted the protein for proteasome-mediated proteolysis. The involvement of GA in this process became clear when Ueguchi-Tanaka and others (2005) demonstrated that *GID1,* loss of which also caused GA-insensitivity and DELLA accumulation in rice, encoded a soluble, nuclear-localised GA receptor with similarity to hormone-sensitive lipases. They showed that association of GA with GID1 promoted interaction with SLR1, the rice DELLA protein. On the basis of domain analysis and mutagenesis experiments, Ueguchi-Tanaka and others (2007) proposed a molecular model, later confirmed by the X-ray crystal structure of GID1 (Shimada and others 2008) and of an *Arabidopsis* ortholog AtGID1a (Murase and others 2008), whereby binding of GA (GA_4_ was the most effective GA) in a pocket allowed the flexible N-terminal strand of GID1 to associate with the top of the pocket, acting as a lid. This conformational change is necessary for interaction with the DELLA protein, which occurs through this protein’s DELLA and VHYNP motifs. The interaction with GID1-GA promotes DELLA’s association with the F-box protein and hence its degradation (Griffiths and others 2006), although the details of this process at the molecular level are still unclear. Rice contains a single GID1 receptor, whereas *Arabidopsis* has three paralogs (Nakajima and others 2006), with considerable redundancy such that loss of a single paralog has no effect on the phenotype, while the two double knockouts produce different phenotypes and loss of all three receptors results in a very extreme GA-insensitive dwarf (Griffiths and others 2006). This redundancy may explain why GID1 was discovered in rice rather than *Arabidopsis*, in which mutant screens for the receptor were unsuccessful.

The establishment of DELLA proteins as key components of GA signalling has focused research on DELLA function and down-stream events. It is known that they regulate gene expression with as many genes activated as suppressed (Zentella and others 2007). They do not contain a recognisable DNA-binding domain, but act in association with transcription factors. The first reported examples of a direct association with transcription factors were the independent demonstrations by two groups that DELLAs interact with PHTOCHROME INTERACTING FACTORs (PIFs) in the *Arabidopsis* hypocotyl and thereby prevent their activation of gene expression (de Lucas and others 2008; Feng and others 2008). However, apart from this sequestration of transcription factors, DELLAs have also been shown to act as co-activators of gene expression through interaction with INDETERMINATE-type transcription factors (Yoshida and others 2014). A recent example of this is the interaction of GAI with GAI-ASSOCIATED FACTOR1 (GAF1) (Fukazawa and others 2014). Intriguingly in association with DELLA protein, GAF1 promotes expression of GA-biosynthetic genes that are subject to feedback regulation so providing the molecular basis for this regulation and the accumulation of GAs in DELLA gain-of-function mutants, as observed by Radley 45 years ago. A number of DELLA partners are components of signalling pathways for other hormone classes, as, for example, the transcription factors BZR1, involved in brassinosteroid signalling (Gallego-Bartolome and others 2012), and JAZ in jasmonate signalling (Hou and others 2010), indicating the high degree of cross-talk between GA signalling and these pathways.

## Gibberellin Transport

The early application experiments indicated that GA_3_ was mobile in plants and the first studies to investigate GA transport, such as that by Kato (1958) in which he measured movement through pea stems between agar blocks, established that GA transport, unlike that of auxin, was non-polar, with equal movement in acropetal and basipetal directions. To enable detection, Kato used very high amounts of GA, but subsequent experiments by several groups with radiolabelled GAs at physiological concentrations confirmed the non-polar nature of GA transport in shoot tissue sections, although there was evidence for polar, basipetal movement from root tips (reviewed in Jacobs and Jacobs 2001). The rate of movement was much less than for polar auxin transport. Based on the observed inhibition of [^3^H]GA_1_ movement through oat coleoptiles by sodium azide, Drake and Carr (1979) concluded that GA transport is symplastic, occurring via plasmodesmata. This accorded with the “ion trap” model in which the weakly acidic GAs are ionised in the alkaline environment of the cytosol and unable to diffuse through the plasma membrane, whereas in the more acidic apoplast, they would be protonated and rapidly taken up into cells. Kramer (2006) estimated that the decay length of GAs in the apoplast and xylem would be measured in micrometers, with 13-hydroxylated GAs surviving slightly longer in this environment. O’Neill and others (1986) had reached a similar conclusion based on the high permeability of GA_1_ in cowpea membrane vesicles, and predicted it would be translocated efficiently in the weakly alkaline phloem. They suggested also that accumulation of GA_1_ in the cytosol would disrupt the membrane pH gradient and stressed the importance of metabolism to more polar metabolites that could be stored in the vacuole. This group had previously suggested from work with leaves and protoplasts from cowpea and barley that GA_1_ is converted by 2β-hydroxylation to GA_8_, which was compartmentalised in the vacuole, mainly as the glucoside (Garcia-Martinez and others 1981). Indeed, Musgrave and others (1971) had suggested earlier that accumulation of [^3^H]GA_1_ in barley aleurones was associated with metabolism to more polar products.

What is the physiological relevance of GA transport? On the basis of the co-location of genes encoding GA-biosynthetic enzymes and signalling components, Kaneko and others (2003) concluded that GAs are synthesised at their site of action in shoot apices and stamens of rice. However, some organs are dependent on an external source of GAs, notable examples being the cereal aleurone, which receives GA from the embryo scutellum (Lenton and others 1994), and petals, which are dependent on the anthers as their GA source (Weiss and Halevy 1989). Long distance transport of GAs from leaves has been implicated in floral initiation at the shoot apex in a number of species (King 2012), and in the promotion of elongation and secondary growth of the stem (Dayan and others 2012; Garcia-Martinez and Rappaport 1982). Rescue of GA-deficient mutants in grafting experiments has also demonstrated long distance movement of GAs, and while grafting between wild-type and mutant maize seedlings implied movement of bioactive GA (Katsumi and others 1983), experiments with pea and potato indicated that the precursor GA_20_ rather than GA_1_ was the mobile form (reviewed in Ross and others 2006). Recently, grafting experiments with *Arabidopsis* mutants provided clear evidence that GA_12_ is the main mobile form in this species in both the xylem and phloem (Regnault and others 2015). These grafting experiments demonstrate that leaves and roots are capable of providing GAs and/or precursors to support the growth of shoots. However, as shoots would normally be autonomous for GA, the physiological relevance of these observations needs clarification.

The identification of GA-like substances in phloem and xylem exudates (Hoad and Bowen 1968; Reid and others 1969) is consistent with GAs being transported by both these routes. However, as discussed above, while phloem transport of GAs would be predicted, transport in the xylem is not consistent with the ion trap model based on passive diffusion of the neutral molecules through membranes. Furthermore, on the basis of scanning colorimetry and electron spin resonance experiments with artificial phospholipid membranes, Pauls and others (1982) concluded that GA_4_ and GA_7_ associate with the membrane surface, but do not penetrate. Transport of GAs would therefore appear to require trans-membrane transporters, particularly efflux transporters, which would also fit with the apparent high structural specificity of the GAs that are transported (Regnault and others 2015). The recent report that GA-fluorescein conjugates accumulated in the endodermis of *Arabidopsis* roots is evidence also of cellular specificity (Shani and others 2013). GA transporters are now being identified, although they lack specificity and are capable of transporting other hormones as well as unrelated molecules (Chiba and others 2015; Saito and others 2015). It is anticipated that further GA transporters will be found in the near future.

## Evolution of Gibberellin Biosynthesis and Signal Transduction

The availability of genome sequences for numerous organisms has prompted interest in the evolution of GA production and signalling. The lycophyte *Selaginella moellendorffii*, but not the bryophyte *Physcomitrella patens*, contains functional GA-biosynthesis and signalling pathways, indicating that they evolved in vascular plants (Hirano and others 2007; Vandenbussche and others 2007; Yasumura and others 2007). In *Selaginella*, GA signalling regulates sporulation, but not growth, and it is suggested that the pathway evolved to regulate GAMYB, which is involved in reproductive development even in less advanced plants such as *Physcomitrella* (Aya and others 2011). The development of a role for GA in growth responses in higher plants may have occurred through modifications to DELLA that extended the range of transcription factors with which it can interact. Gibberellin production has evolved in some fungal and bacterial species and, at least in fungi, this seems to have occurred independently of that in plants (Hedden and others 2002). *G. fujikuroi* is now known to consist of a number of mating populations, the rice pathogen being reclassified as *Fusarium fujikuroi* (Leslie 1999). Members of this species complex have distinct plant hosts, and many have lost the capability to produce GAs through mutation and/or loss of parts of the GA-biosynthesis gene cluster (Malonek and others 2005), perhaps indicating that GA production is no longer beneficial to the fungus. On the other hand, GA production is present in a number of distantly related species (Kawaide and Sassa 1993; Rademacher and Graebe 1979), and may have been passed between fungal species by gene transfer. Gibberellins have no known physiological function in fungi, which secrete GAs to modify their host plants, with evidence that they may compromise the plant’s defence mechanism by interfering with jasmonate signal transduction (Hou and others 2010; Navarro and others 2008). Some bacteria also produce GAs, the nitrogen-fixing endophyte *Bradyrhizobium japonica*, for example, is capable of producing GA_9_ (Mendez and others 2014), although there is as yet no indication of function.

## Present and Future

Since the first experiments in the late 1950s, the chemistry, biochemistry and genetics of GA biosynthesis have been resolved to a considerable extent. Nevertheless, a few unsolved questions remain. For example, an alternative GA 20-oxidase that converts the lactone form of the C-20 alcohol to the aldehyde (Ward and others 1997) is likely to make a major contribution to GA biosynthesis, but has not been characterised. Furthermore, the precise mechanism by which C-20 is lost is still unresolved. Although GA 13-hydroxylases have been identified in rice as cytochrome P450s, other enzymes with this activity must be present, as mutants lacking both GA13ox paralogs are not completely deficient in 13-hydroxy GAs (Magome and others 2013). The regulation of GA biosynthesis by developmental and environmental factors is an area of considerable current interest, and the recent progress in understanding the molecular mechanism for GA homeostasis at the transcriptional level is an important advance. However, work suggesting that GA feedback regulation may also operate at the level of protein stability (Lee and Zeevaart 2007) needs to be followed up. Research on GA signalling is focussed on identifying the transcription factors with which DELLA proteins associate to activate or suppress gene expression, as well as their gene targets. A non-transcriptional mechanism for DELLA was reported in the regulation of microtubule assembly, through nuclear sequestration by DELLA of the chaperone component Prefoldin5 (Locascio and others 2013). By enabling microtubule assembly and orientation in the cytosol, GA promotes the transverse orientation of microfibrils, producing the anisotropic cell growth characteristic of GA action (Shibaoka 1993). As well as alternative DELLA functions, there remains the question of whether GA signalling can occur independently of DELLA, as has been suggested for GA-mediated fruit growth in *Arabidopsis* (Fuentes and others 2012).

Mapping precisely the sites of GA biosynthesis and action in plants is an essential prerequisite for understanding how GA signalling is regulated. The sensitivity of physicochemical methods for analysing GA concentrations, GC–MS and more recently liquid chromatography–mass spectrometry has improved enormously, but is still not sufficient for measuring the concentrations of GAs and precursors at the cellular level. The development of in situ methods for identifying the cells that produce, accumulate and respond to bioactive GAs is an important objective as is the further characterisation of GA transporters. Although the GA field has developed immeasurably in the last 100 years, there is still considerable scope for further advances.
